# Whole exome sequencing reveals novel variants associated with diminished ovarian reserve in young women

**DOI:** 10.3389/fgene.2023.1154067

**Published:** 2023-03-29

**Authors:** Na Li, Wanxue Xu, Huimin Liu, Rui Zhou, Siqi Zou, Shiqing Wang, Siyu Li, Zexin Yang, Yongjun Piao, Yunshan Zhang

**Affiliations:** ^1^ School of Medicine, Nankai University, Tianjin, China; ^2^ Department of Obstetrics and Gynecology, Center for Reproductive Medicine, Peking University Third Hospital, Beijing, China; ^3^ Center for Reproductive Medicine, Department of Obstetrics and Gynecology, Peking University Third Hospital, Beijing, China; ^4^ Key Laboratory of Assisted Reproduction, Ministry of Education, Beijing, China; ^5^ Beijing Key Laboratory of Reproductive Endocrinology and Assisted Reproduction, Beijing, China; ^6^ Graduate school, Tianjin Medical University, Tianjin, China; ^7^ Department of Center for Reproductive Medicine, Tianjin Central Hospital of Obstetrics and Gynecology, Tianjin, China

**Keywords:** ovarian reserve, whole exome sequencing, female infertility, point mutation, molecular etiology

## Abstract

**Background:** Diminished ovarian reserve is one of the most important causes of female infertility. In the etiology study of DOR, besides age, it is known that chromosomal abnormality, radiotherapy, chemotherapy and ovarian surgery can result in DOR. For young women without obvious risk factors, gene mutation should be considered as a possible cause. However, the specific molecular mechanism of DOR has not been fully elucidated.

**Methods:** In order to explore the pathogenic variants related to DOR, twenty young women under 35 years old affected by DOR without definite factors damaging ovarian reserve were recruited as the research subjects, and five women with normal ovarian reserve were recruited as the control group. Whole exome sequencing was applied as the genomics research tool.

**Results:** As a result, we obtained a set of mutated genes that may be related to DOR, where the missense variant on GPR84 was selected for further study. It is found that GPR84^Y370H^ variant promotes the expression of proinflammatory cytokines (TNF-α, IL12B, IL-1β) and chemokines (CCL2, CCL5), as well as the activation of NF-κB signaling pathway.

**Conclusion:** In conclusion, GPR84^Y370H^ variant was identified though analysis for WES results of 20 DOR patients. The deleterious variant of GPR84 could be the potential molecular mechanism of non-age-related pathological DOR through its role in promoting inflammation. The findings of this study can be used as a preliminary research basis for the development of early molecular diagnosis and treatment target selection of DOR.

## Graphical Abstract

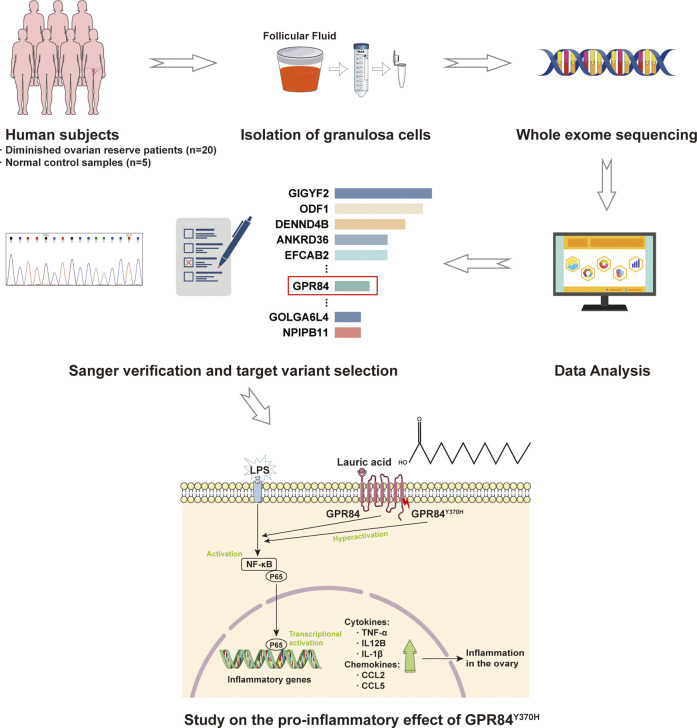

## Introduction

Ovarian reserve, refers to the remaining follicles or oocytes in the ovaries, which is not infinite for a woman from birth. Diminished ovarian reserve, commonly known as DOR, which is characterized by reduction of follicle pool, sometimes along with menstrual abnormalities, ovulation dysfunction and reduced organ, and systemic function owing to low estrogen levels in severe cases. The incidence of DOR ranges from 6% to 64% among infertile women of different ages, which is increasing in recent years (Hu et al., 2020). The assessment of one’s ovarian reserve includes antral follicle count (AFC), serum level of anti-Müllerian hormone (AMH), follicle-stimulating hormone (FSH), inhibin B and so on. In 2020, the Practice Committee of the American Society for Reproductive Medicine (ASRM) suggested that currently AMH and AFC would be the most sensitive and reliable markers of ovarian reserve (Practice Committee of the American Society for Reproductive Medicine. Electronic address and Practice Committee of the American Society for Reproductive Medicine, 2020). The diagnostic criteria of DOR are not uniform among different clinics across the world. According to the Bologna criteria suggested by ESHRE in 2011, DOR is diagnosed when serum level of AMH <0.5–1.1 ng/mL and AFC < 5–7, which has been the reference of all criteria since then.

DOR is an important cause of female infertility since the depletion of follicle is reported to be associated with ovulation disorder. In assisted reproductive technology for infertile women, DOR indicates poor prognosis because it may result in poor ovarian response (POR) to ovarian stimulation during IVF/ICSI-ET. The pathological mechanism of DOR can be divided into two types: a smaller initial follicle pool and accelerated depletion of follicles (Jaswa et al., 2021). It is well established that ovarian reserve decreases with age. Over the total lifetime, a woman’s ovarian reserve will certainly meet the criteria for DOR when approaching menopause (Greene et al., 2014). It is called “Age-related DOR” when age is the sole factor contributing to DOR (Liu et al., 2021). Other than this physiological condition, there are various etiology factors that accelerate the depletion of follicles, affect ovarian reserve, and thus bring about pathologic DOR, including genetic abnormity, diseases causing ovarian injury such as endometriosis and pelvic infection, iatrogenic factors such as ovarian surgery, chemotherapy and pelvic irradiation, environmental and lifestyle factors such as smoking (Collaborators, 2021).

Among all the causes, genetic factors appear to be important and have not been fully elucidated. So far it has been reported that chromosome abnormality (e.g., 45, X mosaicism), gene mutation [e.g., *FMR1* premutation (Tang and Yu, 2020)], polymorphisms [*GDF9* (Wang et al., 2013), *FSHR* (Livshyts et al., 2009), and *ESR1* (Livshyts et al., 2013)], differential gene expression [e.g., *AMH*(Skiadas et al., 2012) and epigenetic effects (Nilsson et al., 2012)] were related to the disease. In many cases, DOR is a multifactorial disease, but some idiopathic cases occurring in young women may have an underlying genetic component (Greene et al., 2014). It is of great significance to explore the genetic etiology of DOR for the early identification and intervention. Novel sequencing technologies have been substantial for expediting our understanding of the genetics of diseases. Over the past decade, the emerging next-generation sequencing technology (NGS) has shown its promising and efficient application in genetic study of human reproduction diseases. In order to explore the underlying genetic factors involved in DOR, whole exome sequencing (WES) is an ideal approach since it only focuses on the exons or protein coding regions in the genome and is more cost-effective than whole genome sequencing (WGS).

In the sample selection, most of the previous studies used peripheral blood as the sample source (Wang et al., 2019; Liu et al., 2020; Rouen et al., 2022), which could only detect genetic variations, while somatic mutations will not be detected. The most ideal experimental sample in our study would be oocytes, but the oocytes of patients are so precious that they must be reserved for subsequent *in vitro* fertilization and cannot be used as sequencing samples. In the ovaries, every oocyte and the surrounding granulosa and theca cells form the functional unit-the follicle, which provides a protective and suitable environment for the oocyte. Ovarian granulosa cells (GCs) are situated closely adjacent to the oocyte and there are plentiful and various material exchange and signal transduction between them. Furthermore, GCs are the synthesis sites of vital hormones including estrogen and AMH, and thus play a key role in folliculogenesis and ovarian function (Strauss and Williams, 2019). Many studies have suggested that the apoptosis or dysfunction of GCs has a negative effect on the quantity or quality of oocytes, thus resulting in ovarian insufficiency. Therefore, mural GCs in follicular fluid which are readily available in IVF/ICSI procedure may be an appropriate choice for studying the genetic abnormalities especially somatic mutation associated with DOR. Somatic mutations occurring in GCs will have a great impact on ovarian reserve, so we proposed to use ovarian GCs as the sequencing sample in order to find out more significant variants.

In order to identify deleterious variants occurring in young DOR patients without other obvious pathogenic factors, so as to provide clues and basis for the early identification and risk prediction of DOR, we enrolled 20 DOR patients who underwent IVF/ICSI in the reproductive center of Tianjin Central Hospital of Gynecology Obstetrics, and performed whole exome sequencing of the ovarian granulosa cells collected from the patients to determine causal genetic variances of DOR.

## Materials and methods

### Ethical approval

The research was approved by the reproductive ethics committee of Tianjin Central Hospital of Gynecology Obstetrics (No. ZY2021001, 06/01/2021), and the study was conducted in accordance with the declaration of Helsinki. All patients had given their informed consent for the use of their samples for future researches.

### Human subjects

From May 2018 to September 2022, of all age groups there were 382 DOR patients without definite factors impacting ovarian reserve, out of 8,715 patients undergoing IVF/ICSI-ET in the reproductive center of Tianjin Central Hospital of Gynecology Obstetrics. From these females, twenty women affected by DOR and five women with normal ovarian reserve were recruited in the study. The clinical assessment and definition of DOR in our study were based on the Bologna criteria. DOR was diagnosed when the serum level of AMH ≤1.1 ng/mL and AFC < 5-7, while matched women with normal ovarian reserve were included into the control group. All the included cases were younger than 35 years old and had a normal karyotype of 46, XX. Females with FMR1 premutation were excluded, as were patients with PCOS, history of ovarian surgery, pelvic infections, chemotherapy, pelvic radiation, autoimmune disease, and other severe medical conditions.

### Sample preparation

Primary ovarian GCs were isolated refer to red blood cell lysis protocol for RBC lysis as described below. Patients’ follicular fluid collected on the day of oocyte pick-up during the IVF/ICSI procedure was centrifuged at 450 × g for 10 min at room temperature. Decant the supernatant and resuspend the cell pellet in 3 mL of PBS. Add 30 mL of red blood cell lysis buffer (Solarbio Science & Technology, Beijing, China), and then invert to mix. Incubate on ice for 15 min, during which invert gently every 5 min. Centrifuge at 450 × g for 6 min at 4°C and decant the supernatant. Resuspend the pellet gently in 20 mL of red blood cell lysis buffer and pellet GCs by centrifugation at 450 × g for 6 min at 4°C.

### Genomic DNA extraction, isolation and whole exome sequencing

Total genomic DNA was extracted and isolated by conventional phenol-chloroform extraction. Before sequencing, the concentration of DNA was measured by Qubit Fluorometer and the integrity and purification were detected by Agarose Gel Electrophoresis. Library preparation and sequencing were carried out following certified protocols of the Beijing Genomics Institute (BGI). Briefly, the genomic DNA was randomly fragmented and adapters were added to both ends of the fragments followed by PCR amplification. The qualified library from each sample was respectively loaded on BGISEQ-500 sequencing platforms to perform high-throughput sequencing. The raw image files produced by the sequencer were processed using BGISEQ-500 base caller with default parameters, and the sequencing data were stored in FASTQ files.

### Filtering, mapping, and local realignment

The raw reads were preprocessed as follows: 1) The reads containing adapters were removed; 2) The reads containing more than 50% low-quality bases (base quality ≤5) were filtered; 3) The reads containing more than 10% N bases were removed for the downstream analysis. The high-quality reads were mapped to the human reference genome (GRCh38) using Burrows-Wheeler Aligner (BWA v0.7.15) (Li and Durbin, 2010) and duplicates were marked and discarded *via* Picard-tools (v2.5.0). Then, the reads were subjected to local realignment around insertions/deletions (indels) and base quality score recalibration (BQSR) using the Genome Analysis Toolkit (GATK v3.7) (McKenna et al., 2010) following the best practice pipeline (DePristo et al., 2011).

### Variant calling, annotation, and filtering

Single-nucleotide variants (SNVs) and indels were called using GATK3.7 (HaplotypeCaller) and annotated with SnpEff software (Cingolani et al., 2012). To filter irrelevant variants, we selected only variants whose predicted impact on the protein were “high” or “moderate”. The variants with minor allele frequency (MAF) ≥ 5% in 1,000 Genomes database were removed. The pathogenicity scores of the variants were calculated using various packages, including SIFT (Ng and Henikoff, 2003), Mutation assessor (Schwarz et al., 2010), PolyPhen2 (Adzhubei et al., 2013), and MetaSVM(Zaucha et al., 2020), and the variants with SIFT score ≤0.05 or PolyPhen2 ≥ 0.909 or Mutation Assessor score ≥1.9 or MetaSVM = “deleterious” were selected for downstream analysis.

### Sanger verification

Approximately 400–800 bp DNA fragments containing the selected variant site were amplified by PCR using high fidelity DNA polymerase KOD-Plus (Toyobo, Osaka, Japan) and the primers in Supplementary Table S3 according to the manual. PCR products were purified by 1% agarose gel and gel Midi Purification Kit (Tiangen, Beijing, China) and sequenced by Sanger sequencing on ABI 3730XL sequencers at Sangon Biotech (Shanghai, China). DNA sequences were aligned with NCBI by BLASTN programs and Chromas software.

### Cell culture

KGN cells were purchased from Procell Life Science&Technology (Wuhan, China). Cells were cultured in DMEM/F12 medium (Biological Industries, USA) supplemented with 10% FBS (Biological Industries) and 100 IU/mL penicillin/streptomycin (Gibco, Grand Island, NY) in a humidified atmosphere of 5% CO_2_ at 37°C.

### Construction of stable cell lines

The full-length coding sequence and mutated sequence of human *GPR84* was amplified by PCR using GCs’ cDNA as template from normal females. The sequences were respectively cloned into plasmid pLV-EF1α-MCS-IRES-Bsd (Biosettia). All plasmids were validated by Sanger sequencing. The procedures of lentivirus vectors package in HEK293 cells and infection were described previously (Chang et al., 2015). Cells infected with lentivirus were screened with 2.5 μg/mL of blasticidin (Thermo Fisher Scientific, Waltham, MA).

### RNA extraction and real-time quantitative PCR (qRT-PCR)

Total RNA was extracted with Trizol reagent (Thermo Fisher Scientific) according to the protocol, and reversely transcribed with Hifair^®^ II 1st Strand cDNA Synthesis Kit (Yeasen Biotech, Shanghai, China). Real-time quantitative polymerase chain reaction (qRT-PCR) analysis was performed using SYBR Green SuperMix (Yeasen Biotech) on LightCycler96 system (Roche, Basel, Switzerland). The primer sequences are listed in Supplementary Table S4. The relative gene expression was calculated by the 2^−ΔΔCt^ method with β-actin as a loading control.

### Protein extraction and Western blot

Cells were lysed in RIPA lysis buffer with protease inhibitor cocktail (Roche) and phosphatase inhibitor cocktail (Sigma-Aldrich, St Louis, MO). The protein concentration was measured by BCA Protein Assay Kit (Thermo Fisher Scientific). The same amount of protein was loaded into SDS-PAGE gels for electrophoresis, and transferred onto a PVDF membrane (Merck, Darmstadt, Germany). After blocking with 5% defatted milk for 1 h at room temperature, the membrane was incubated with primary antibodies: GPR84 (1:2500, Invitrogen, Waltham, MA, USA), NF-κB p65 (1:1,000, CST, Danvers, MA, USA), β-actin (1:5000, Affinity Biosciences), α-tubulin (1:1,000, CST), Lamin A/C (1:2500, Proteintech, Wuhan, China) at 4°C overnight. The HRP-conjugated secondary antibodies (goat anti-rabbit IgG, goat anti-mouse IgG (1:2500, ZSGB-BIO, Beijing, China) were using for incubation of the membranes for 1 h at room temperature. The signals were visualized with an ECL detection system and photographed by Tanon-5200 (Tanon, Shanghai, China). The relative protein level was quantified with Image J 1.48v (NIH, Bethesda, MD).

### Statistical analysis

Statistical analysis was performed in Prism 8.0.1 (GraphPad Software, San Diego, CA, USA). Quantitative data were presented as means ± SEM, and the differences among the groups were analyzed by Student’s two-tailed *t*-test. Differences are considered statistically significant at *p < 0.05; **p < 0.01; ***p < 0.001 and ****p < 0.0001; ns means no significance.

## Results

### Clinical information of included subjects

The baseline characteristics of included women were summarized in Supplementary Tables S1, S2. There were no significant differences in baseline characteristics such as age, BMI, and infertility period between the DOR and control group.

### Landscape of variations in DOR

**FIGURE 1:**
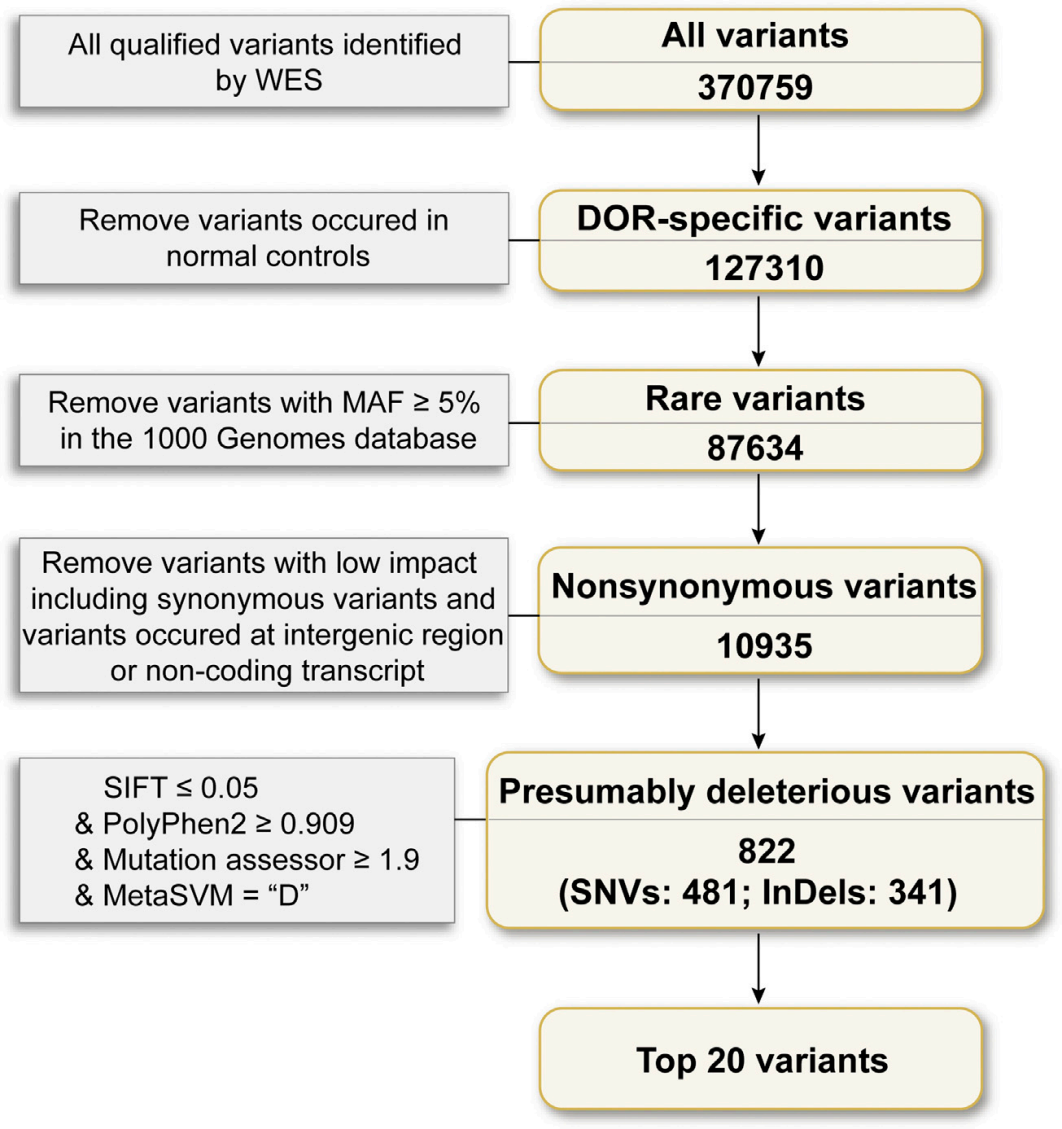
Framework of WES data analysis. Of the qualified variants, the variants 1) identified in normal samples; 2) MAF >5% in the 1,000 Genome database; 3) in the non-coding regions; 4) predicted as no impact were removed, and top 20 mutated variants were finally reported.

To explore the landscape of variations related to DOR, we performed WES of 20 DOR cases and 5 controls who underwent IVF/ICSI in the reproductive center of Tianjin Central Hospital of Gynecology Obstetrics. On average, 231 million reads passed quality control for each sample, and the mean sequencing depth on target regions was 235.89×. On average per sequencing individual, 98.86% of the targeted bases had at least 10× coverage. To filter irrelevant variations, we designed a systemic variation selection framework as shown in Figure 1. Briefly, an average number of 370,759 variants passed the GATK quality control in each individual, including 313,744 SNVs and 57,015 indels. Firstly, the variants overlapped with those identified in the normal samples were removed, and 127,310 variants left, after which the 87,634 variants with MAF <5% in the 1,000 Genomes database were chosen for the downstream analysis. Next, the non-synonymous SNVs and frameshift indels were selected followed by further choosing variants identified as deleterious using various pathogenicity prediction software, including SIFT ≤0.05, Polyphen2 ≥ 0.909, Mutation assessor ≥1.9 and MetaSVM = “Deleterious”.

**FIGURE 2:**
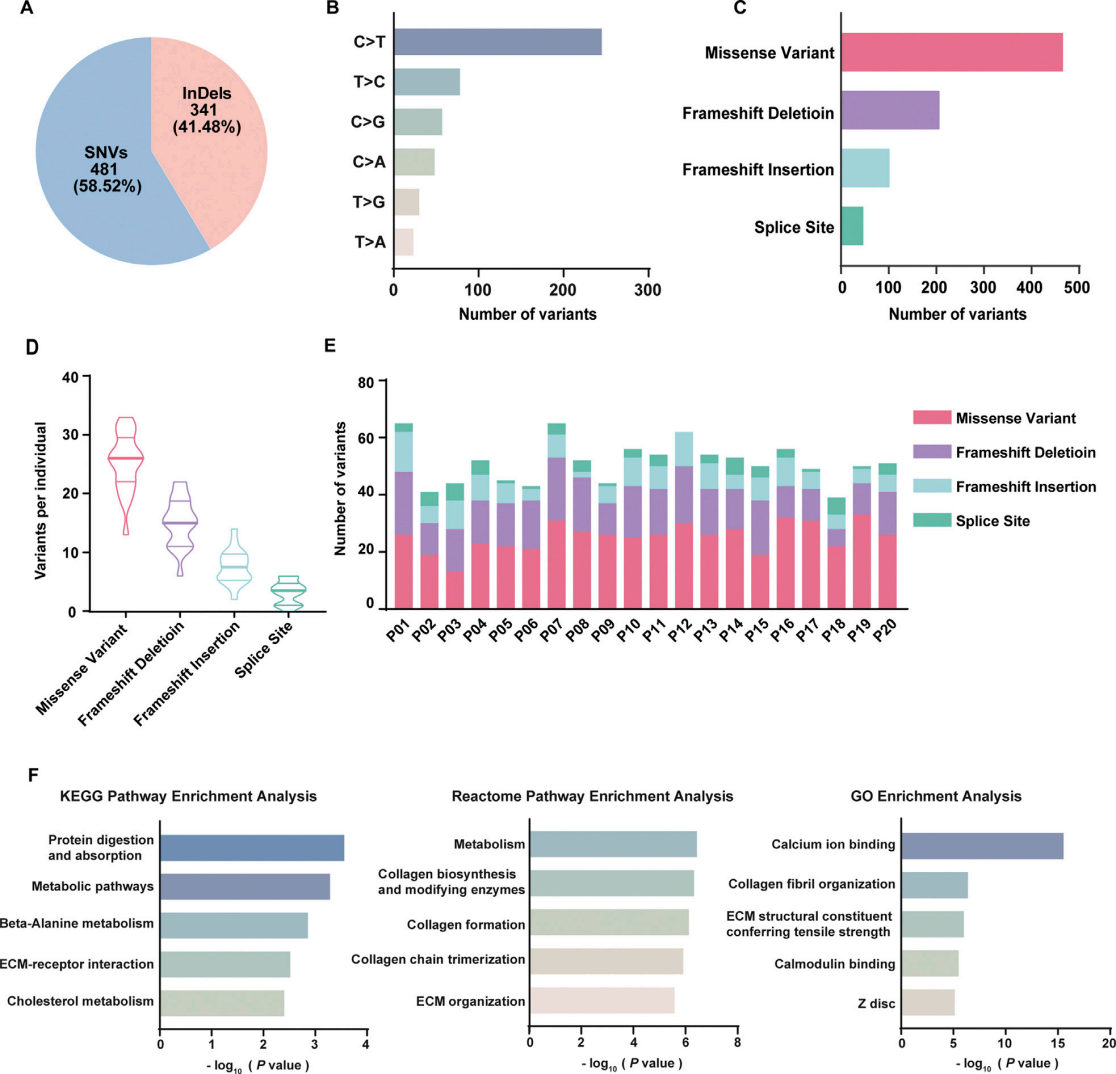
Summary of whole exome sequencing results of 20 DOR patients. **(A)** The percentage of SNVs and indels in filtered variants; **(B)** The classes of SNVs and their frequencies; **(C)** The frequencies of different types of variants in total samples; **(D)** The violin plot of the number of different types of variants in each patient; **(E)** The distribution of different types of variants in each patient; **(F)** Functional analysis of 731 genes using KEGG, Reactome, and GO database.

As a result, 822 variants involving 731 genes were identified related to DOR, including 481 (58.52%) SNVs and 341 (41.48%) indels (Figure 2A). Of 481 SNVs (Figure 2B), 258 were C>T (49.33%), 89 were T>C (17.02%), 61 were C>G (11.66%), 52 were C>A (9.94%), 36 were T>G (6.88%), 27 were T>A (5.16%). In total, the variants included 466 missense variants, 15 SNVs at splice site, 207 frameshift deletion, 102 frameshift insertion, and 32 indels at splice site (Figure 2C). On average for each individual, there were 25 missense variants, 15 frameshift deletions, 8 frameshift insertions and 3 variants at splice site (Figure 2D). The distribution of different types of variants in each patient was shown in Figure 2E. High-throughput technologies, such as WES, can generate a large list of genes of interest. However, it is difficult to extract biological meaning from the list due to the noisy nature of biological processes. To explore the underlying relevance among these mutated genes, we conducted pathway enrichment analysis and functional annotation through KEGG database (Kanehisa et al., 2019), Reactome database (Gillespie et al., 2022) and Gene Ontology (GO) database (Ashburner et al., 2000; Gene Ontology, 2021) (Figure 2F). It was found that the mutated genes were enriched in KEGG pathways including protein digestion and absorption, metabolic pathways, beta-Alanine metabolism, ECM-receptor interaction, and cholesterol metabolism; the genes were enriched in Reactome pathways including metabolism, collagen biosynthesis and modifying enzymes, collagen formation, collagen chain trimerization, and ECM organization. The GO analysis revealed the mutated genes may be involved in calcium ion binding, collagen fibril organization, ECM structural constituent conferring tensile strength, calmodulin binding, and Z disc.

### Top mutated variants in DOR

**FIGURE 3:**
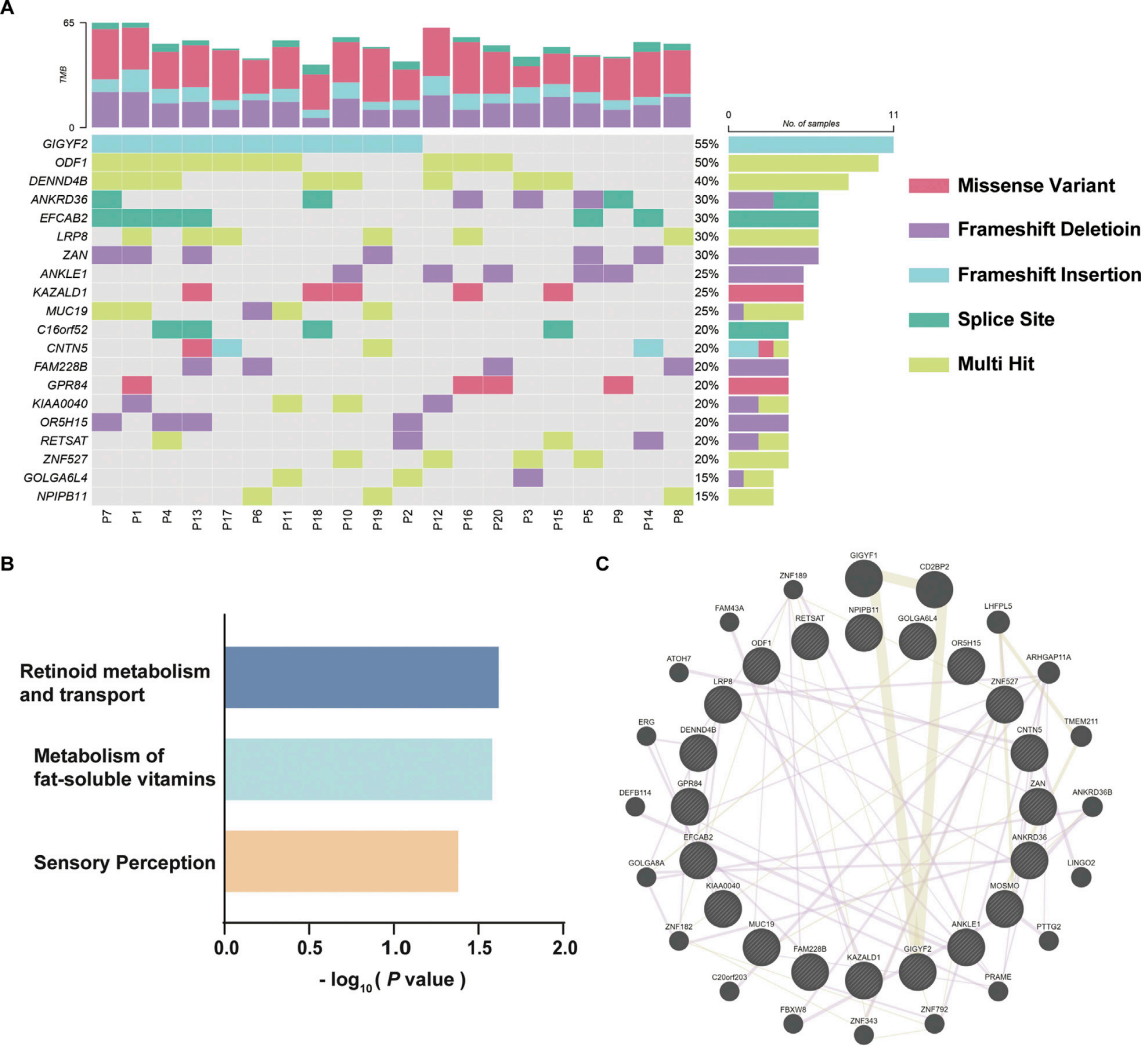
Top 20 mutated genes in DOR. **(A)** The variant characteristics of top 20 mutated genes in each patient. The twenty DOR patients included for WES were represented by “P01”-“P20”. **(B)** Enrichment analysis of top 20 mutated genes using Reactome pathways. **(C)** Protein-protein interactions of mutated genes identified using GENEMANIA database.

To identify reliable variants related to DOR, the 731 mutated genes were sorted according to the variation frequency in 20 samples, and the top 20 mutated genes were finally reported, which were *GIGYF2*, *ODF1*, *DENND4B*, *ANKRD36*, *EFCAB2*, *LRP8*, *ZAN*, *ANKLE1*, *KAZALD1*, *MUC19*, *C16orf52*, *CNTN5*, *FAM228B*, *GPR84*, *KIAA0040*, *OR5H15*, *RETSAT*, *ZNF527*, *GOLGA6L4*, and *NPIPB11* (Figure 3A). *GIGYF2* has the highest prevalence of amino acids altering variants identified in our DOR cohort with frameshift insertions of GC in the same genomic position of mutated samples. *KAZALD1* and GRP84 only contained missense variants; *ODF1*, *DENND4B*, *LRP8*, *ZNF527* and *NPIPB11* had multiple variants in the genes; *ANKRD36*, *MUC19*, *CNTN5*, *KIAA0040*, *RETSAT* and *GOLGA6L4* had mixed types of variants in different samples. The details of the identified variants in top 20 genes were listed in Table 1. To explore the underlying relevance of top 20 mutated genes, we conducted pathway enrichment analysis and functional annotation (Figure 3B). It was found that the mutated genes were enriched in Reactome pathways including retinoid metabolism and transport, metabolism of fat-soluble vitamins, and sensory perception. Then, we investigated the interactions among these mutated genes using GENEMANIA database (Warde-Farley et al., 2010), as shown in Figure 3C. There were connections such as co-expression or protein-protein interaction among these genes which were reported before, although not clearly elucidated yet.

**TABLE 1:** The variants of top 20 mutated genes.

Gene	Frequency of variation	Variant type	chr	Variant
*GIGYF2*	11/20 (55%)	Frameshift_insertion	chr2	NM_001103147.1:p.Gln1232fs
				/c.3692_3693insGC
*ODF1*	10/20 (50%)	Frameshift_deletion	chr8	NM_024410.3:p.Ser222fs
				/c.665_672delGCCCCTGC
		Frameshift_deletion	chr8	NM_024410.3:p.Asn225fs
				/c.674_683delACCCGTGCAG
*DENND4B*	8/20 (40%)	Frameshift_insertion	chr8	NM_014856.2:p.Gln902fs
				/c.2703_2704insG
		Frameshift_insertion	chr8	NM_014856.2:p.Gln902fs
				/c.2702_2703insGC
*ANKRD36*	6/20 (30%)	Frameshift_deletion	chr2	NM_001164315.1:p.Ser1154fs
				/c.3461_3462delCT
		Splice_variant	chr2	NM_001164315.1:c.3956-3dupT
*EFCAB2*	6/20 (30%)	Splice_variant	chr1	NM_001143943.1: c.374-6_374-2dupTTTTA
*LRP8*	6/20 (30%)	Frameshift_deletion	chr15	NM_004631.4:p.Gln25fs
				/c.73_74delCA
		Frameshift_deletion	chr1	NM_004631.4:p.Leu24fs
				/c.71delT
*ZAN*	6/20 (30%)	Frameshift_deletion	chr7	NM_003386.2:p.Cys 1923fs
				/c.5768delG
*ANKLE1*	5/20 (25%)	Frameshift_deletion	chr19	NM_001278444.1:p.Leu645fs
				/c.1933_1942delTTGTGTGTGT
*KAZALD1*	5/20 (25%)	Missense_variant	chr10	NM_030929.4:p.Cys76Gly
				/c.226T>G
*MUC19*	5/20 (25%)	Frameshift_deletion	chr12	NM_173600.2:p.Lys5212fs
				/c.15635delA
		Frameshift_insertion	chr12	NM_173600.2:p.Thr5971fs
				/c.17911dupA
		Frameshift_deletion	chr12	NM_173600.2:p.Arg5500fs
				/c.16498_16511delAGGGCTACCTGGAG
*C16orf52*	4/20 (20%)	Splice_variant	chr16	NM_001164579.1: c.106 + 1_106+2insAGCACTCAC
*CNTN5*	4/20 (20%)	Frameshift_insertion	chr11	NM_001243270.1:p.Phe89fs
				/c.264_265insAACTGAGGAACCAGGCATTATTTTGTCGATAGATCCAAAATTGACAAAGGTAGACAACATCTAGAAAATATTA
		Missense_variant	chr11	NM_001243270.1:p.Arg244Trp
				/c.730C>T
		Missense_variant	chr11	NM_001243270.1:p.Tyr1041Cys
				/c.3122A>G
*FAM228B*	4/20 (20%)	Frameshift_deletion	chr2	NM_001145710.1:p.Gln303fs
				/c.907delC
*GPR84*	4/20 (20%)	Missense_variant	chr12	NM_020370.2:p.Tyr370His
				/c.1108T>C
		Missense_variant	chr12	NM_020370.2:p.Pro348Leu
				/c.1043C>T
*KIAA0040*	4/20 (20%)	Frameshift_deletion	chr1	NM_001162893.1:p.Lys69fs
				/c.204delG
		Frameshift_deletion	chr1	NM_001162893.1:p.Asn65fs
				/c.195_202delCAAGAAGA
*OR5H15*	4/20 (20%)	Frameshift_deletion	chr3	NM_001005515.1:p.Ser103fs
				/c.307delT
*RETSAT*	4/20 (20%)	Frameshift_deletion	chr2	NM_017750.3:p.Phe134fs
				/c.402delT
		Frameshift_insertion	chr2	NM_017750.3:p.Tyr468fs
				/c.1400_1401insAC
		Frameshift_deletion	chr2	NM_017750.3:p.Thr466fs
				/c.1397_1398delCT
*ZNF527*	4/20 (20%)	Frameshift_insertion	chr19	NM_032453.1:p.Pro301fs
				/c.901_902insTGTG
		Frameshift_deletion	chr19	NM_032453.1:p.Tyr302fs
				/c.904delT
*GOLGA6L4*	3/20 (15%)	Frameshift_deletion	chr15	NM_001267536.2.2:p.Asp164fs
				/c.489delA
*NPIPB11*	3/20 (15%)	Frameshift_deletion	chr16	NM_001310137.1:p.Glu520fs
				/c.1557delT
		Frameshift_deletion	chr16	NM_001310137.1:p.Glu478fs
				/c.1431_1555del
		Frameshift_deletion	chr16	NM_001310137.1:p.Asp535fs
				/c.1605delT
		Frameshift_deletion	chr16	NM_001310137.1:p.Asp493fs
				/c.1479_1603del

### GPR84^Y370H^ variant promotes the inflammatory response in human ovarian granulosa cells

**FIGURE 4:**
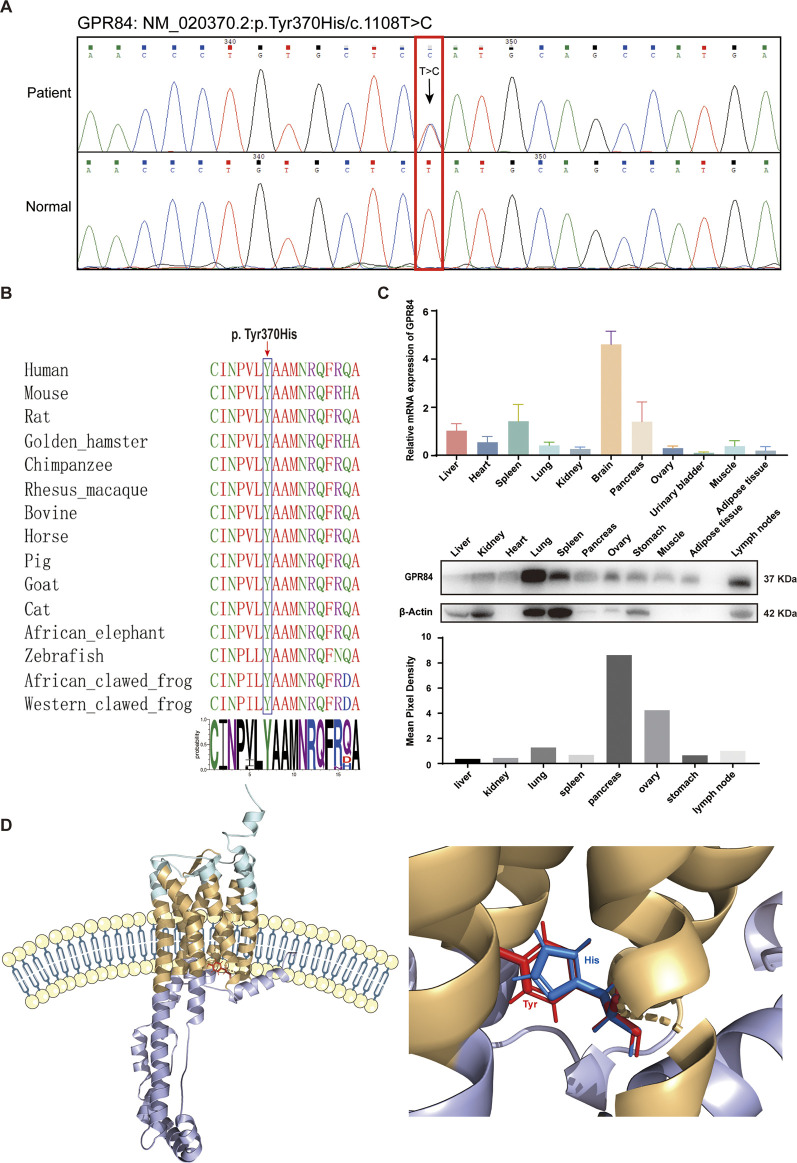
Characteristics of the GPR84 variant. **(A)** Sanger sequencing validation of the heterozygous c.1108T > C variant in the *GPR84* gene. The red box indicates the variant site. **(B)** Amino acid sequence alignment of GPR84 among different species. The blue box indicates the mutated amino acid. Tyrosine at position 370 is 100% conserved across all species. **(C)** The expression of GPR84 in different tissue and organs in BALB/c mouse were measured by qRT-PCR and Western blot. **(D)** Location of the mutated amino acid in the GPR84 protein predicted by RoseTTAFold. The extracellular segments are shown in cyan; the cytoplasmic segments are shown in violet; the transmembrane segments are shown in yellow. The wildtype amino acid tyrosine is shown in red, and the mutated amino acid histidine is shown in blue.

According to the Sanger sequencing results and biological functions of the top mutated genes, the missense variant (NM_020370.2:p.Tyr370His/c.1108T>C) in GPR84 was selected as the target for further study. The characteristics of *GPR84* variant were summarized in Figure 4. The missense variants detected in *GPR84* by Sanger sequencing were exactly the same as the WES results (Figure 4A). To rule out polymorphism or genetic instability at the variant site, we performed amino acid sequence alignment of GPR84 among different species. As shown in Figure 4B, tyrosine at position 370 is 100% conserved across all species, which suggested that the variant occurred at this site may be pathogenic. In order to see the expression of GPR84 in ovaries which has not been reported in databases or researches, we detected the mRNA and protein level of GPR84 in different tissue and organs in BALB/c mouse. The results showed that GPR84 was expressed in the ovary to some extent (Figure 4C), so we believe that it can be further studied as a research target for the etiology of DOR. To determine the exact location and feature of the variant site, we predicted the protein structure of GPR84 by RoseTTAFold (Baek et al., 2021). The results showed that the variant site was located within the cell membrane, close to the intracellular site. The electrically neutral, hydrophobic amino acid tyrosine of a six-membered ring mutates to the positively charged, hydrophilic histidine of a five-membered ring (Figure 4D).

**FIGURE 5:**
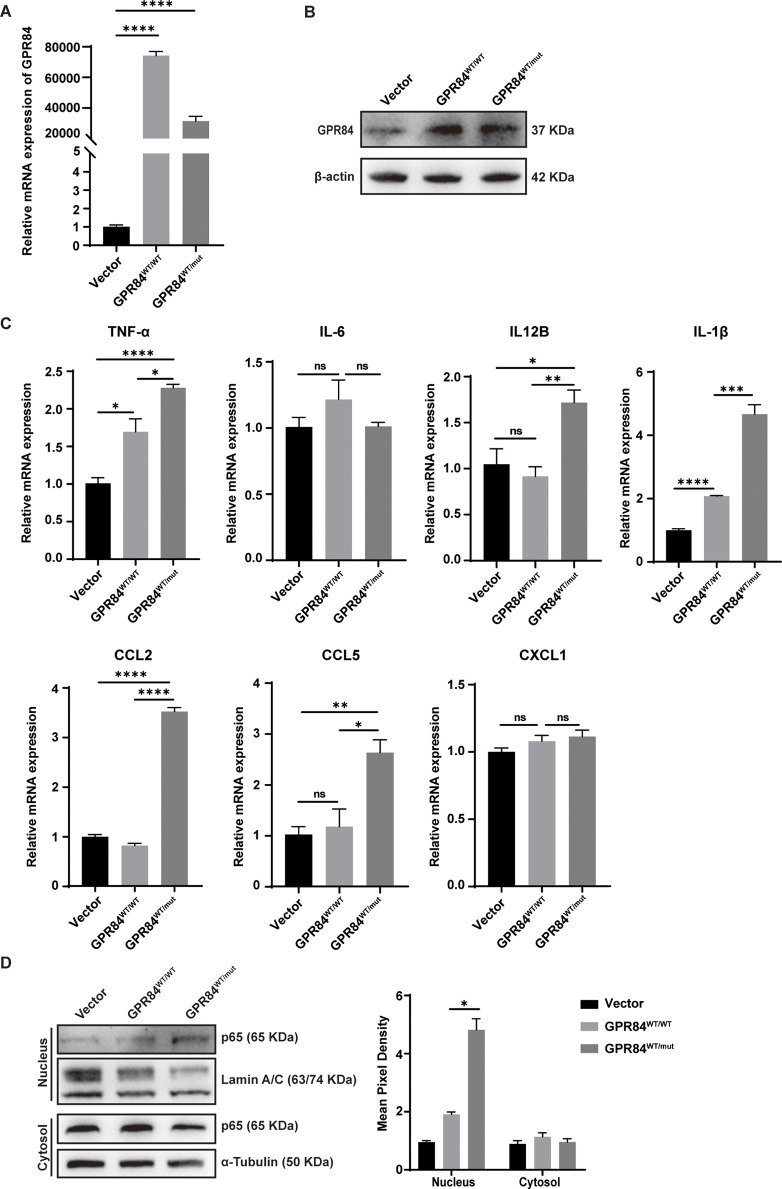
GPR84^Y370H^ variant promotes the inflammatory response in human ovarian granulosa cells. **(A, B)** The construction of GPR84 (shown as GPR84^WT/WT^) and GPR84^Y370H^ (shown as GPR84 ^WT/mut^) overexpressed KGN cells (human granulosa-like tumor cell line) were verified by qRT-PCR and Western blot respectively. **(C, D)** The GPR84^WT/WT^ and GPR84^WT/mut^ overexpressed KGN cells were treated with LPS (0.1 μg/mL) for 2 h before stimulation with lauric acid (200 μM) for 24 h. **(C)** The mRNA level of inflammatory cytokines (TNF-α, IL-6, IL12B, IL-1β) and chemokines (CCL2, CCL5, CXCL1) were measured by qRT-PCR. **(D)** The protein level of p65 in nucleus were increased in GPR84^WT/mut^ group compared with GPR84^WT/WT^ group. Lamin A/C and α-Tubulin were used as loading controls in nucleus and cytosol respectively. Data are represented as mean ± SEM. Statistical significance was assessed by two-tailed unpaired *t*-test. *p < 0.05, **p < 0.01, ***p < 0.001, ****p < 0.0001.

To further explore the effect of GPR84^Y370H^ variant on GCs, we constructed the GPR84^Y370H^ overexpressed (shown as GPR84^WT/mut^) and GPR84 overexpressed (shown as GPR84^WT/WT^) stable cell lines in KGNs (human granulosa-like tumor cell line), while the GPR84^WT/WT^ group was regarded as the control (Figures 5A, B). GRP84 was reported as an enhancer of inflammatory signaling (Recio et al., 2018b). To study the influence of GPR84^Y370H^ variant on its proinflammatory effect, KGNs were pretreated with LPS (0.1 μg/mL) for 2 h to set up an inflammation environment before stimulation with GPR84’s biological ligand lauric acid (200 μM) for 24 h. The mRNA level of inflammatory cytokines (TNF-α, IL-6, IL12B, IL-1β) and chemokines (CCL2, CCL5, CXCL1) were measured by qRT-PCR. As shown in Figure 5C, GPR84^Y370H^ variant promotes the expression of TNF-α, IL12B, IL-1β, CCL2 and CCL5. In order to explore the effect of GPR84^Y370H^ variant on the activation of inflammation-associated NF-κB signaling pathway, we conducted nuclear and cytoplasmic protein extraction on the cells that have been treated as described above, and tested NF-κB subunit p65 in nucleus and cytosol by Western blot. The results showed that the protein level of p65 in nucleus were increased in GPR84^WT/mut^ group compared with GPR84^WT/WT^ group (Figure 5D), which indicated that GPR84^Y370H^ variant promoted the activation of NF-κB.

## Discussion

In addition to age, factors such as history of ovarian surgery (Zhao et al., 2020), radiotherapy, chemotherapy (Fenton, 2015), and endometriosis (Muzii et al., 2018; Ashrafi et al., 2019) can lead to the decline of female ovarian reserve. However, there still are many young females who have no definite factors damaging ovarian function also suffer from diminished ovarian reserve, resulting in infertility, poor ovarian response in IVF, adverse pregnancy outcomes and so on. DOR in young women is easily neglected, which can bring about serious and irreversible impacts on female fertility. Therefore, it is of great significance to screen out susceptible population of DOR at the early stage so that they can get guidance on fertility timing and necessary treatment as soon as possible. Premature ovarian insufficiency (POI) is the loss of normal ovarian function before 40 years old (Fenton, 2015), which has a similar pathological basis with DOR. It has been speculated that the two diseases share some etiological mechanism in common (Zhao et al., 2019). With the rapid development of next-generation sequencing technology, the association of POI etiology with genetic mutations has been supported by many researches (Jiao et al., 2018), and numerous potential pathogenic mutations have been found (Qin et al., 2015; Jaillard et al., 2020; Chon et al., 2021). However, there are still few studies on the genetic molecular etiology of DOR.

Here, we performed whole exome sequencing in 20 young DOR patients and 5 women with normal ovarian reserve to explore the potential genetic mechanism of DOR. In the sample selection, most of the previous studies used peripheral blood as the sample source (Wang et al., 2019; Liu et al., 2020; Rouen et al., 2022), which could detect the genomic variation, but the somatic mutation occurred locally in the ovary will not be detected. The most ideal experimental sample should be oocytes, but the oocytes of patients are so precious that they must be reserved for subsequent *in vitro* fertilization and cannot be used as sequencing samples. It is confirmed that GCs play a key role in oocyte development, follicular growth and maturation (Strauss and Williams, 2019). Somatic mutations occur in GCs will have a great impact on ovarian reserve, so we proposed to use ovarian GCs as the sequencing sample in order to find out more significant variants.

Though a series of filtering steps for WES data, we identified 822 functional deleterious variants involving 731 genes. Pathway enrichment analysis and GO functional annotation showed that most of the genes were enriched in pathway such as the biosynthesis and metabolism of ECM, collagen, calcium, and lipid, which are associated with ovarian function. The variants in the top 20 genes with highest variant frequencies among the 20 samples were verified by Sanger sequencing. It is confirmed that the reported variants do occur in *ODF1*, *EFCAB2*, *ZAN*, *KAZALD1* and *GPR84*. Through literatures reviewing, it is found that most of these genes had not been reported as related to ovarian reserve previously. By studying their basic biological functions and verifying the variants though Sanger sequencing, the missense variant in the gene *GPR84* was selected as the target for further study.

G protein-coupled receptor 84 (GPR84), also known as inflammation-related G protein-coupled receptor EX33, is a member of metabolite-sensing G protein-coupled receptors (GPCRs) family with medium-chain fatty acid (MCFA) as its natural ligand (Recio et al., 2018a; Luscombe et al., 2020). GPR84 functions as an enhancer of inflammatory signaling in macrophages once inflammation is established, characterized by enhanced expression of phosphorylated Akt, p-ERK, and p65 nuclear translocation and elevated levels of the inflammatory mediators TNFα, IL-6, IL-12B, CCL2, CCL5, and CXCL1 (Recio et al., 2018b; Wojciechowicz and Ma’ayan, 2020). In our study, it is found that GPR84^Y370H^ variant enhanced inflammation response in ovarian granulosa cells under proinflammatory stimulation. It has been reported that chronic low-grade inflammation is a major contributor to the pathogenesis of premature ovarian insufficiency (Agacayak et al., 2016; Huang et al., 2019). Therefore, it can be speculated that the variants of GPR84 in ovarian GCs would affect ovarian reserve through its role in promoting inflammation, and thus become a potential etiological molecular mechanism of DOR.

There are still some limitations of the study. Firstly, many cases of POI are familial and hereditary, but the heredity of DOR, which is more common in clinics, has not been clearly reported. In this study, only sporadic DOR patients’ samples were collected for sequencing, while no parental DNA samples were obtained, so it is difficult to determine whether the variant is hereditary or not. Secondly, the definite impacts and mechanisms of GPR84^Y370H^ variant on GCs, oocytes and follicles need thorough study *in vitro* and *in vivo* to determine whether they can really impair ovarian reserve. Thirdly, whole exome sequencing only focuses on the protein coding region and the junction between exons and introns, whereas the role of non-coding regulatory regions needs other researches to study.

In conclusion, we conducted WES on 20 young females with DOR and obtained a set of mutated genes related to DOR, where the GPR84^Y370H^ variant was identified. The deleterious variants of GPR84 could be the potential molecular mechanism of non-age-related pathological DOR through its role in promoting inflammation. The findings of this study can be used as a preliminary research basis for the development of early molecular diagnosis and treatment target selection of DOR.

## Data Availability

The data presented in the study are deposited into the Sequence Read Archive database, accession number PRJNA943550.
