# Supplementary material for: Whole exome sequencing reveals novel variants associated with diminished ovarian reserve in young women
Source: Front Genet. 2023 Mar 29;14:1154067. doi: 10.3389/fgene.2023.1154067 (PMC10095150; doi:10.3389/fgene.2023.1154067)
Supplement: Supplementary file 1 [file Table1.DOCX]

**Supplementary Material**

Table S1. Baseline characteristics of included patients

| Characteristics | DOR group (N=20) | Control group (N=5) | *P* value |
| --- | --- | --- | --- |
| Age (years) | 30.80 ± 2.63 | 30.80 ± 1.48 | 1.000 |
| BMI (kg/m^2^) | 22.61 ± 3.24 | 22.61 ± 1.55 | 0.403 |
| Infertility period (years) | 3.35 ± 1.73 | 3.80 ± 2.78 | 0.649 |
| AMH^a^ (ng/mL) | 0.61 ± 0.34 | 4.00 ± 1.58 | 0.008 |
| Basal FSH^b^ (mIU/mL) | 8.63 ± 2.71 | 6.15 ± 0.97 | 0.089 |
| Basal LH^c^ (mIU/mL) | 4.89 ± 7.79 | 6.80 ± 1.52 | 0.637 |
| Basal E_2_^d^ (pg/mL) | 45.28 ± 15.70 | 37.75 ± 9.98 | 0.373 |
| Basal T^e^ (ng/dL) | 26.84 ± 13.11 | 27.20 ± 20.93 | 0.962 |

^a^AMH: anti-Müllerian hormone; ^b^FSH: follicle-stimulating hormone; ^c^LH: luteinizing hormone; ^d^E_2_: estradiol; ^e^T: testosteron
