# Supplementary material for: Whole exome sequencing reveals novel variants associated with diminished ovarian reserve in young women
Source: Front Genet. 2023 Mar 29;14:1154067. doi: 10.3389/fgene.2023.1154067 (PMC10095150; doi:10.3389/fgene.2023.1154067)
Supplement: Supplementary file 2 [file Table2.DOCX]

**Supplementary Material**

Table S2. Clinical characteristics of included DOR patients

| Patients | Age (years) | BMI  (kg/m^2^) | Infertility period  (years) | Menstrual period  (days) | Menstrual  cycle  (days) | Basal AMH (ng/mL) | AFC | Basal FSH (mIU/mL) | Basal LH  (mIU/mL) | Basal E_2_  (pg/mL) | Basal T  (ng/dL) |
| --- | --- | --- | --- | --- | --- | --- | --- | --- | --- | --- | --- |
| P01 | 27 | 22 | 2 | 6 | 35-90 | 0.6 | <5-7 | 10.7 | 1.7 | <20 | 19 |
| P02 | 32 | 22 | 4 | 6-7 | 28 | 0.9 | <5-7 | 5.1 | 0.9 | 32.1 | 35.8 |
| P03 | 33 | 25.5 | 2 | 5-6 | 28 | 0.5 | <5-7 | 6.4 | 4.4 | 54.2 | 30.7 |
| P04 | 32 | 27.6 | 1 | 6 | 30 | 0.4 | <5-7 | 4.1 | 1.1 | 36.1 | 26 |
| P05 | 34 | 21 | 6 | 6 | 27 | 0.9 | <5-7 | 9.7 | 1.9 | 52.3 | 14.6 |
| P06 | 34 | 18.8 | 7 | 6 | 32 | 1.1 | <5-7 | 7.8 | 3.4 | 47.8 | 19.7 |
| P07 | 33 | 18.6 | 7 | 4-5 | 29 | 0.8 | <5-7 | 10.5 | 6.4 | 20 | 24.7 |
| P08 | 29 | 23 | 3 | 5 | 24-25 | 0.9 | <5-7 | 10 | 2.9 | 42.3 | 21.3 |
| P09 | 32 | 24.1 | 2 | 3-4 | 31-32 | 1.1 | <5-7 | 8.4 | 1.4 | 34.8 | 17.4 |
| P10 | 33 | 18.8 | 3 | 6-7 | 28-29 | 0.3 | <5-7 | 4 | 1.6 | 38.6 | 23.9 |
| P11 | 31 | 24.1 | 4 | 7 | 28 | 0.69 | <5-7 | 10.88 | 3.55 | 31.94 | 23 |
| P12 | 27 | 23.4 | 2 | 7 | 30 | 0.3 | <5-7 | 11.2 | 3 | 27.8 | 19.6 |
| P13 | 29 | 19.1 | 2 | 4 | 30 | 0.7 | <5-7 | 8.7 | 6.1 | 77.3 | 45.8 |
| P14 | 32 | 29 | 2 | 7 | 28-30 | 0.8 | <5-7 | 6.2 | 3.5 | 52.2 | 73.8 |
| P15 | 33 | 18.4 | 3 | 3-5 | 25 | 0.6 | <5-7 | 5.9 | 3.6 | 55.9 | 22.1 |
| P16 | 29 | 23.9 | 4 | 6 | 23 | 0.7 | <5-7 | 7.4 | 2.3 | 53.3 | 19.3 |
| P17 | 30 | 18.4 | 5 | 3 | 25 | 0.04 | <5-7 | 12.3 | 5.92 | 78.5 | 20 |
| P18 | 28 | 24.4 | 2 | 3-4 | 60-90 | <0.01 | <5-7 | 125 | 37.3 | 28.5 | 29.8 |
| P19 | 33 | 27 | 3 | 5-10 | 35-90 | 0.06 | <5-7 | 12.35 | 3.82 | 55.61 | 25.81 |
| P20 | 25 | 23 | 3 | 5-6 | 50 | 0.9 | <5-7 | 8.5 | 3.1 | 41 | 24.5 |
| Average | 30.8 | 22.61 | 3.35 | - | - | 0.647 | - | 14.26 | 4.895 | 45.28 | 26.84 |
