# Supplementary material for: Whole exome sequencing reveals novel variants associated with diminished ovarian reserve in young women
Source: Front Genet. 2023 Mar 29;14:1154067. doi: 10.3389/fgene.2023.1154067 (PMC10095150; doi:10.3389/fgene.2023.1154067)
Supplement: Supplementary file 3 [file Table3.DOCX]

**Supplementary Material**

Table S3. Primers for PCR of fragment containing variant site

| Primers’ name | Sequence (5’→3’) | Product size (bp) |
| --- | --- | --- |
| *ODF1*-Variant-forward | TGTGATTCGCATGGGTGGAC | 586 |
| *ODF1*-Variant-reverse | GGCTTCCACAGGGATAACACG |  |
| *EFCAB2*-Variant-forward | GCTGTCCAGCGTCAGCAAAT | 549 |
| *EFCAB2*-Variant-reverse | AGAGAAGAGAACAGCTAGACAAAGC |  |
| *ZAN*-Variant-forward | CCCACATGTTTGGGGTCAGG | 464 |
| *ZAN*-Variant-reverse | GCTCTTGGTTGCAGCAGAGT |  |
| *KAZALD1*-Variant-forward | TGACCTCCTGACCTGGCTTC | 511 |
| *KAZALD1*-Variant-reverse | GAGTAGGTGTGACCGTCGGA |  |
| *GPR84*-Variant-forward | GCATCAGGAGGACCCAGTGA | 503 |
| *GPR84*-Variant-reverse | GGTCCTGGAGGAGACAGTCC |  |
| *GPR84*-CDS-forward | TCAGGATCCGCCACCATGTGGAACAGCTCTGACGCCAAC | 1191 |
| *GPR84*-CDS-reverse | TCAGCTAGCCTAATGGAGCCTATGGAAACTC |  |
| *GPR84^Y370H^-forward* | ACCCTGTGCTCCATGCAGCCATGAACCGCCAATTCC | 1191 |
| *GPR84^Y370H^-reverse* | ATGGCTGCATGGAGCACAGGGTTGATGCAACCA |  |
