# Supplementary material for: Whole exome sequencing reveals novel variants associated with diminished ovarian reserve in young women
Source: Front Genet. 2023 Mar 29;14:1154067. doi: 10.3389/fgene.2023.1154067 (PMC10095150; doi:10.3389/fgene.2023.1154067)
Supplement: Supplementary file 4 [file Table4.DOCX]

**Supplementary Material**

Table S4. Primers for qRT-PCR of genes involved in the study

| Primers’ name | Sequence (5’→3’) | Product size (bp) |
| --- | --- | --- |
| *GPR84*-forward | CATCCAGCCCAAGCTCCGTA | 219 |
| *GPR84*-reverse | TAGCGTCCCAGTGCGATGAG |  |
| *TNF-α*-Forward | TGGAGAAGGGTGACCGACTC | 268 |
| *TNF-α*-Reverse | TCCCAGGTTTCGAAGTGGTGG |  |
| *IL6*-Forward | CCTTCGGTCCAGTTGCCTTC | 239 |
| *IL6*-Reverse | TTCTGCCAGTGCCTCTTTGC |  |
| *IL12B*-Forward | GCTGGGAGTACCCTGACACC | 213 |
| *IL12B*-Reverse | GCACAGATGCCCATTCGCTC |  |
| *IL-1β*-Forward | CTTCCTGGGAGGGACCAAAGG | 202 |
| *IL-1β*-Reverse | CAGGCAGTTGGGCATTGGTG |  |
| *CCL2*-Forward | AGCAAACCCAAACTCCGAAGA | 274 |
| *CCL2*-Reverse | AACATCCCAGGGGTAGAACTG |  |
| *CCL5*-Forward | TCCTGTATGACTCCCGGCTG | 261 |
| *CCL5*-Reverse | TGTAACTGCTGCTGTGTGGTAGA |  |
| *CXCL1*-Forward | AAAGATGCTGAACAGTGACAAATCC | 272 |
| *CXCL1*-Reverse | AGGACAGTGTGCAGGTAGAGTTA |  |
